# Plasma Lipoproteins as Mediators of the Oxidative Stress Induced by UV Light in Human Skin: A Review of Biochemical and Biophysical Studies on Mechanisms of Apolipoprotein Alteration, Lipid Peroxidation, and Associated Skin Cell Responses

**DOI:** 10.1155/2013/285825

**Published:** 2013-04-23

**Authors:** Paulo Filipe, Patrice Morlière, João N. Silva, Jean-Claude Mazière, Larry K. Patterson, João P. Freitas, R. Santus

**Affiliations:** ^1^Faculdade de Medicina de Lisboa, Hospital de Santa Maria, Clínica Dermatologica Universitaria, Avenida Professor Egas Moniz, 1699 Lisboa Codex, Portugal; ^2^INSERM, U1088, UFR de Pharmacie, 3 rue des Louvels, 80036 Amiens, France; ^3^CHU Amiens, Pôle Biologie, Pharmacie et Santé des Populations, Centre de Biologie Humaine, Laboratoire de Biochimie, Avenue René Laennec, Salouël, 80054 Amiens, France; ^4^UFR de Médecine et de Pharmacie, Université de Picardie Jules Verne, 3 rue des Louvels, 80036 Amiens, France; ^5^Radiation Laboratory, University of Notre Dame, Notre Dame, IN 46556, USA; ^6^Département RDDM, Muséum National d'Histoire Naturelle, 43 rue Cuvier, 75231 Paris, France

## Abstract

There are numerous studies concerning the effect of UVB light on skin cells but fewer on other skin components such as the interstitial fluid. This review highlights high-density lipoprotein (HDL) and low-density lipoprotein (LDL) as important targets of UVB in interstitial fluid. Tryptophan residues are the sole apolipoprotein residues absorbing solar UVB. The UVB-induced one-electron oxidation of Trp produces ^•^Trp and ^•^O_2_^−^ radicals which trigger lipid peroxidation. Immunoblots from buffered solutions or suction blister fluid reveal that propagation of photooxidative damage to other residues such as Tyr or disulfide bonds produces intra- and intermolecular bonds in apolipoproteins A-I, A-II, and B100. Partial repair of phenoxyl tyrosyl radicals (TyrO^•^) by **α**-tocopherol is observed with LDL and HDL on millisecond or second time scales, whereas limited repair of **α**-tocopherol by carotenoids occurs in only HDL. More effective repair of Tyr and **α**-tocopherol is observed with the flavonoid, quercetin, bound to serum albumin, but quercetin is less potent than new synthetic polyphenols in inhibiting LDL lipid peroxidation or restoring **α**-tocopherol. The systemic consequences of HDL and LDL oxidation and the activation and/or inhibition of signalling pathways by oxidized LDL and their ability to enhance transcription factor DNA binding activity are also reviewed.

## 1. Introduction

Human skin is chronically attacked by deleterious environmental agents such as ultraviolet (UV) light, ionizing radiation, and air pollutants, for example, ozone. These may generate free radicals and other reactive oxygen species (ROS), which—through processes of oxidative stress [1–3]—can aggravate or even cause many skin disorders including skin cancers, cutaneous autoimmune diseases, phototoxicity, and skin aging.

During the last three decades, the incidence of cutaneous cancers due to exaggerated exposure to solar UV radiation has markedly increased as a result of outdoor occupations and sun-bathing habits. The spectral limit for the solar UV radiation reaching the Earth is ~295 nm. The biological effects of UV light have led photobiologists to separate the solar UV spectrum into two domains, namely, UVB (295–315 nm) and UVA (315–390 nm). While UVA mainly produces oxidative stress [3, 4], UVB is responsible for both direct photochemistry of molecules as well as development of oxidative stress. At the cellular level, the two major early events accompanying exposure to UVB light are the induction of DNA damage and lipid peroxidation [5, 6].

Our group—which involves collaboration among dermatologists, biochemists, chemists, and biophysicists from several countries—has spent more than 20 years investigating the molecular and cellular aspects of UV-induced photooxidative stress related to skin pathophysiology. This review deals with several aspects of our contribution to dermatological science and to the photobiology of skin.

A full comprehension of all mediators involved in the response of human skin to UV light requires that the molecular bases of the multifocal biological effects of the UV radiation be understood. Not only epidermal cells but also extracellular skin components have been considered in our studies. For example, we have been interested in the effect of UV light on some components of the interstitial fluid which feeds the dermis and epidermis, as this fluid plays an essential role in mediating the transport of nutrients, hormones, essential proteins, and lipids required for cell growth and differentiation. In addition to studies concerning the deleterious effects on cells and other components of the skin, preventive molecular strategies against oxidative stress have also been developed by searching for new families of antioxidants.

## 2. The Lipoproteins of the Interstitial Fluid: Neglected Mediators of the Action of the UV Radiation on Skin

Early on, it appeared to us that high-density lipoprotein (HDL) and low-density lipoprotein (LDL) are important UVB targets. First, it has been shown that oxidation of some amino acid residues of lipoproteins such as tyrosine (Tyr), tryptophan (Trp), and lysine (Lys) leads to apolipoprotein alterations and lipid peroxidation [7]. Secondly, thanks to their lipid core, lipoproteins have been shown to be natural carriers of the essential lipophilic antioxidants, *α*-tocopherol (*α*TocOH) and carotenoids (Car) [8], which can reduce UVB-induced oxidative damage in skin [9]. Lastly, apolipoproteins are known to contain tryptophan (Trp), the only aromatic residue absorbing solar UVB (but not UVA) susceptible to UVB-induced photooxidation with subsequent formation of reactive indolyl radicals and ROS as primary species [10].

HDL and LDL like most other macromolecular components of blood can cross vessel walls by a process resembling ultrafiltration. Thus, the interstitial fluid feeding epidermal cells can be considered as a serum ultrafiltrate. Therefore, the concentration of serum proteins in this ultrafiltrate is determined by their molecular size [11, 12]. That is, the smaller the lipoprotein size, the greater its concentration in the interstitial fluid as compared to that in serum. Thus, the concentration of apolipoprotein A-1 (apoA-I)—a principle constituent protein of HDL—is about 15 *μ*M, whereas that of the LDL apolipoprotein B100 (apoB100) is on the order of 0.4 *μ*M. Each of apoA-I and apoB100 contains 4 and 37 Trp residues, respectively. As the indole ring of Trp has appreciable molar extinction coefficients of 1500 M^−1^ cm^−1^ at 295 nm and even 510 M^−1^ cm^−1^ at 300 nm, one may conclude that the most significant fraction of UVB light absorbance by lipoproteins occurs in the 120 *μ*M of HDL_3_ Trp (HDL_3_ has 2 apoA-I) compared to the 15 *μ*M of LDL Trp. For comparison, the concentration of the single Trp of albumin in the interstitial fluid is about 160 *μ*M. Consequently, in the ~50 *μ*m thick layer of the epidermis reached by the UVB radiation, the Trp residues of lipoproteins absorb more than half the quantity of light absorbed by albumin. Thus, despite its large concentration, albumin cannot be considered as an effective sunscreen for the lipoproteins. All these data support the contention that lipoproteins must be considered as mediators in the overall effects of UVB on human skin through the specific or unspecific interaction of photochemically oxidized HDL and LDL with human skin cells.

## 3. The UVB Light Absorption by Trp Residues Is Responsible for Lipoprotein Lipid Peroxidation

**Figure 1:**
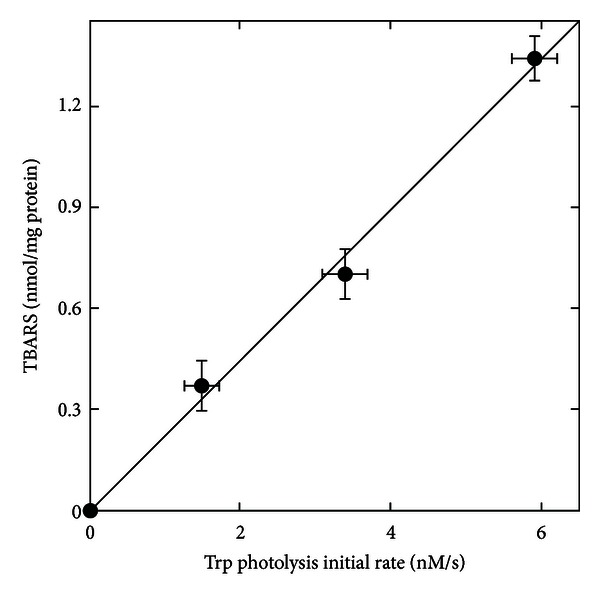
TBARS production as a function of the initial rate of Trp photolysis. TBARS expressed in nmol/mg of protein and the Trp photolysis initial rate expressed in nM/s have been determined with air-saturated pH 7 buffered solutions of HDL at concentrations up to 1 *μ*M. The incident UVB light dose in these experiments was 6.7 J/min. Drawn from data in [14].

Upon irradiation of aerated solutions of LDL and HDL_3_ with UVB light, Trp residues of apoA-I and apoB100 are readily destroyed. The quantum yields of Trp photolysis are 5 × 10^−4^ and 2 × 10^−3^ for LDL and HDL_3_, respectively. This Trp destruction is accompanied by formation of lipid peroxidation decomposition products as measured by the thiobarbituric acid assay (TBARS) and by the consumption of *α*TocOH and Car carried by the lipoproteins. On the other hand, neither Trp destruction nor antioxidant consumption is observed when solutions are irradiated with the UVA radiation [13]. From UVB radiation data in [14], demonstrating that the initial rate of Trp destruction and the corresponding TBARS production are both proportional to the lipoprotein concentration, it can be deduced that TBARS production is proportional to the initial rate of Trp photolysis (Figure 1). Furthermore, since a surfactant will disrupt the lipid molecular organization necessary to lipid peroxidation, 1% SDS was added to the lipoprotein solution before the irradiation in subsequent experiments. This addition did not alter the Trp destruction but suppressed the TBARS production [15]. As a consequence, it is likely that lipid peroxidation in lipoproteins results from the Trp photolysis. The fact that Car are not consumed under UVA irradiation of HDL and LDL solutions—despite strong absorbance in the UVA region—suggests that antioxidant consumption under UVB cannot be solely attributed to direct photobleaching. One must also consider their consumption while acting as inhibitors of ROS and of the “dark” radical chain reactions of the lipid peroxidation. Such chain reactions may be sustained by trace metal ions probably present in the lipoprotein preparations. Accordingly, the strong enhancement of postirradiation damage at the apolipoprotein level by redox metal ions demonstrates a synergism between UVB-induced lipid photoperoxidation and autoperoxidation [13].

The primary process of Trp residue photolysis by UVB radiation in HDL and LDL is undoubtedly the one-electron oxidation (e.g., photoionization) of the indole ring with formation of a neutral Trp radical (^•^Trp) as well as a hydrated electron (*e*_aq_) [10, 15]. In aerated solutions, *e*_aq_ is scavenged by O_2_ at diffusion-controlled rate in competition with reactions involving endogenous electrophilic residues (i.e., in less than 200 ns) to produce the superoxide anion whose dismutation produces H_2_O_2_, making it a primary product of Trp photolysis [10, 16]. An additional demonstration of *e*_aq_ production in one-electron oxidation of Trp residues is the inhibition of 30% of the TBARS upon saturation of the lipoprotein solutions with a mixture of N_2_O/O_2_ (80/20 v/v). This transforms approximately 80% of the *e*_aq_ (hence of ^•^O_2_^−^) into strongly oxidizing ^•^OH radicals. The ^•^OH radicals react nonspecifically with all constituents of HDL or LDL, not only unsaturated lipids but also most residues of apolipoproteins including Trp itself, and a 50% increase of the rate of Trp destruction is observed upon N_2_O/O_2_ saturation [14]. Interestingly, it may be noted that the same ^•^Trp radical has been shown to be involved in the induction of lipid peroxidation, which occurs during the well-established process of Cu^2+^-induced LDL autooxidation. The binding of the redox Cu^2+^ ions in the vicinity of 7 of the 37 apoB100 Trp residues in LDL probably catalyzes the Trp autooxidation [17].

## 4. Propagation of the Photooxidative Trp Damage to Other Apolipoprotein Sites

The major HDL fraction in human serum is that of HDL_3_ composed of two main irregularly distributed proteins: apoA-I (MW: 28 kDa) and apolipoprotein A-II (apoA-II, MW: 17.4 kDa). The remaining proteins comprise less than 10% of the total protein content. On average, 75% of HDL_3_ particles contain 2 apoA-I and 2 apoA-II, while the rest are devoid of apoA-II. The apoA-II is a dimer of 77 residues connected by a disulphide bond with no Trp, free Cys, His, or Arg.

**Figure 2:**
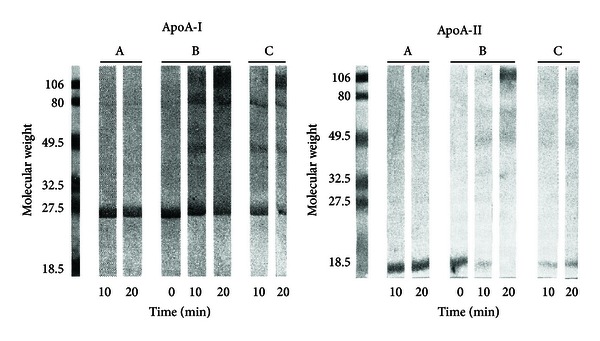
Immunoblots of air-saturated solutions of HDL with antibodies specific for apoA-I or apoA-II. Lanes A: unirradiated samples bubbled with air; lanes B: as in A but irradiated with 6.7 J/minof UVB during the indicated times; lanes C: same as B but the solutions contained 50 *μ*M desferrioxamine, a strong Fe(III) complexing agent. Adapted from [15].

It has been long known that free radical formation in proteins can induce their cleavage or cross-linking [18]. This rule applies to UVB irradiated lipoproteins as shown in Figure 2 which demonstrates that HDL apolipoproteins are strongly altered by such radiation. SDS-polyacrylamide gel electrophoresis and immunoblots with specific monoclonal antibodies reveal that the apolipoprotein alteration, which requires oxygen, occurs after a few minutes of irradiation at low absorbed light (compare lanes A and B in Figure 2). The dose rate was 0.4 J/min with an incident dose rate of 6.7 J/min before complete antioxidant consumption [15]. Self-aggregation of apolipoproteins is a consequence of UVB light absorption by apoA-I Trp residues as unirradiated samples do not form aggregates. Dimers of apoA-I or apoA-II and higher polymers of apoA-I or of both apolipoproteins are also observed. ApoA-II produces dimers although it cannot be directly altered by UVB [15]. Similarly, the formation of high molecular mass apoA-I-containing particles is observed during the Cu^2+^-induced oxidation of dialyzed plasma as obtained with isolated HDL_3_ [19]. Further, both protein modifications and TBARS formation are inhibited upon addition of either desferrioxamine (lanes C; Figure 2), a strong Fe(III) ion complexing agent, or of SDS before irradiation. These effects occurring after only limited Trp photolysis [13, 14] suggest that lipid peroxidation and Fenton-type reactions catalyzed by trace metal ions bound to the lipoproteins play a key role in these alterations. However, the incomplete inhibition of these alterations by desferrioxamine suggests other reaction pathways for the intra- and interapolipoprotein cross-linking. The light-induced polymer formation can result—at least in part—from the propagation of other primary species related to Trp residue photolysis as ^•^Trp can oxidize intact Tyr residues in LDL [20] to produce the tyrosyl phenoxyl radical (TyrO^•^) through long range electron transfer reactions [21]. In HDL, Tyr 100 and Tyr 115 are at sites close to Trp 108 which appears to play a key role in these alternative pathways. Moreover, basic amino acids that react with *e*_aq_ and disulphide bond are split by *e*_aq_, leading to additional radical formation. All these apolipoprotein radical species (noted as ^•^apoA-I, ^•^apoA-II, or ^•^apoB100) can, in turn, contribute to cross-linking and/or polymer formation during Trp photolysis.

As oxidized lipoproteins cannot properly perform their biological functions [22], these results would only be of interest if they were obtained with absorbed light doses of physiological significance. The minimal erythemal doses (MED), that is, the minimum doses required to produce sunburn, for fair-skinned people are about 40 mJ/cm^2^ at 300 nm, 50 mJ/cm^2^ at 304 nm, and 1000 mJ/cm^2^ at 313 nm [23]. Since the *stratum corneum* transmits about 45% and 30% at 313 nm and 304 nm, respectively, the UVB absorbed in the ~50 *μ*m of the epidermal layer [24] at 1 MED around 310 nm is about 50 J/cm^3^. Thus, considering the light doses used in [15], for example, 0.1 to 0.2 J/cm^3^, there is more than enough light at 1 MED to induce lipoprotein alterations in skin similar to those reported in this reference.

**Figure 3:**
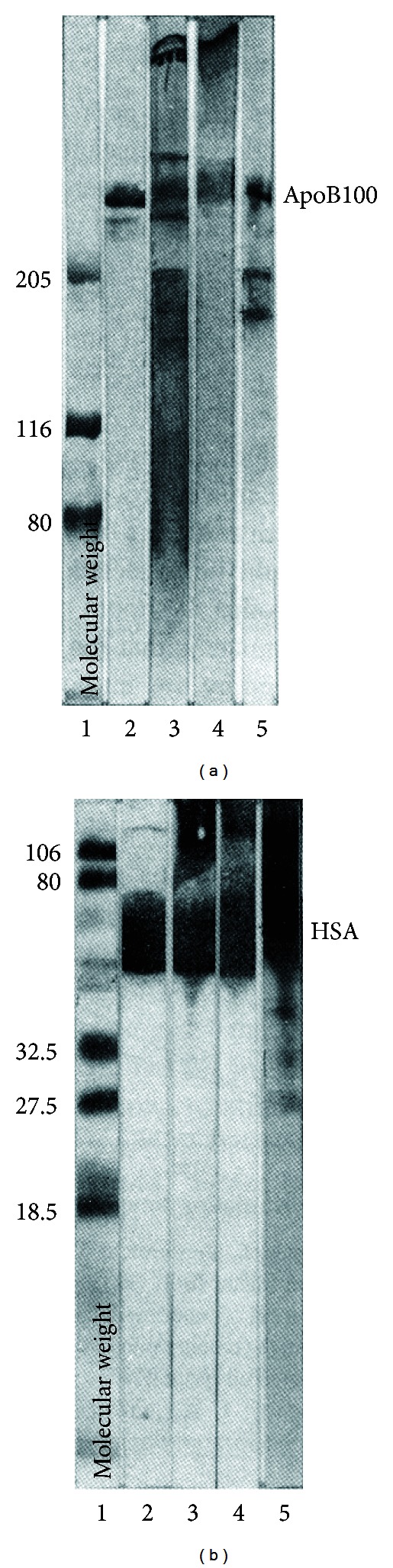
Immunoblots of apoB100 and albumin from air-saturated suction blister fluid before and after irradiation with UVB (absorbed light dose: 12 J/mL). (a) Unmodified apoB100 migration is indicated as apoB100. Lane 1: molecular weight standards; lane 2: unirradiated suction blister fluid (130 *μ*g); lane 3: irradiated suction blister fluid; lane 4: reconstituted blister fluid; lane 5: isolated LDL as reference (prepared from human serum and irradiated). (b) same as (a) but with 20 *μ*g of proteins. See [25] for full experimental details.

A convenient stratagem for gathering the interstitial fluid feeding the dermis and epidermis is to collect blister fluid (0.7 mL) from 2 cm diameter blisters formed by mild suction (−175 mm Hg). In our studies, the methodology followed to demonstrate the oxidative modifications of apolipoprotein by a UVB stress in neutral buffered aqueous solutions has been applied to the suction blister fluid gathered before irradiation [25]. The 8 Trp residues of the two apoA-I of the HDL_3_ fraction and the 37 Trp residues of apoB100 absorb practically 80% as much light as absorbed by the single Trp residue of human serum albumin (HSA). Hence, the HDL and LDL of the suction blister fluid or of a “reconstituted fluid” based on protein concentrations reported in [11, 12] may be readily photooxidized. Such photooxidation leads to a UVB dose-dependent TBARS formation accompanying Trp loss. Furthermore, the same apolipoprotein alterations as those reported with buffered HDL and LDL solutions are observed with appropriate specific monoclonal antibodies as illustrated with apoB-100 of LDL (Figure 3(a)). Incidentally, marked photocleavage and photopolymer formation also occur in blister fluid HSA (Figure 3(b), lane 5) [25]. Although HSA functions as an antioxidant of lipoprotein autooxidation *in vitro* [26] and *in vivo* [27], it is not an effective antioxidant in the photooxidation of the suction blister fluid.

The TBARS formation as well as the structural modifications of apolipoproteins and HSA in the suction blister fluid are induced by UVB irradiation of skin at doses well below 1 MED (see Figure 3 legend). The detection of apoA-II polymers in Figure 3(b) (lanes 3 and 4) suggests again the intervention of radical lipid chain peroxidation reactions which propagate the initial photooxidative damage at the apoA-I level within the HDL particles present in the suction blister fluid. Accordingly, the formation of apoB100 polymers can be attributed to the same reaction sequence [25].

It is of note that, in contrast to buffered solutions of purified lipoproteins [13], irradiation of the suction blister fluid with the UVA radiation produces apoA-I polymers, demonstrating the presence of ROS-producing photosensitizers probably associated with nutrients such as flavins or resulting from nutrient metabolism products in the undialyzed blister fluid.

## 5. Time Course of the Repair of Apolipoprotein Photodamage by Antioxidants

**Figure 4:**
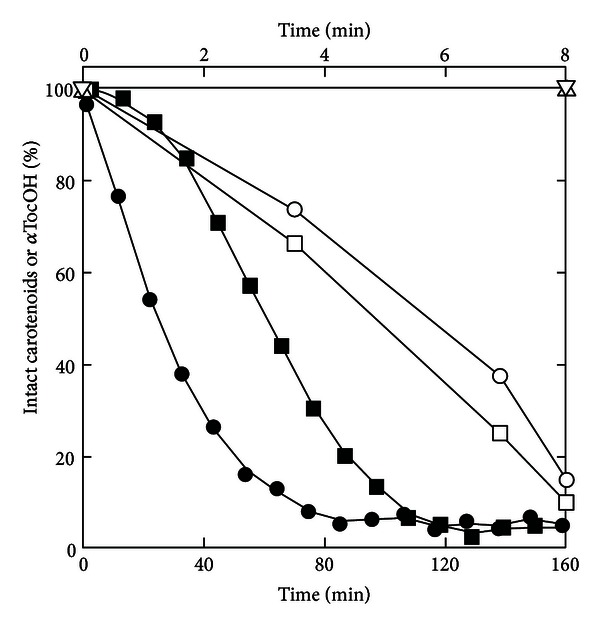
Time courses of Car and *α*TocOH consumption. Lower time scale: carotenoid consumption during Cu^2+^-catalyzed oxidation of 240 nM of LDL in the absence (*⚫*) or presence (■) of 0.75 *μ*M quercetin. Upper time scale: Car (□, ∆) or *α*TocOH (*⚪*,  *▽*) consumption under irradiation of 400 nM of LDL with UVB (□,  *⚪*) or UVA (∆,  *▽*). Drawn from data in [14, 28].

As noted above, the marked consumption of *α*TocOH and Car at low absorbed doses of UVB irradiation cannot be attributed solely to their photobleaching. Although Car strongly absorb UVA (Figure 4), the consumption of Car is arrested immediately by removing the UVB radiation from a UVB + UVA light source. This consumption parallels an immediate production of TBARS [13, 14]. By contrast, time lags of at least 30 min are observed in the Cu^2+^-catalyzed autooxidation of lipoproteins (Figure 4), before TBARS or conjugated diene production which parallels a marked consumption of carotenoids (see also [29]). The notable difference in behavior between the UVB-induced photooxidation and the Cu^2+^-induced autoperoxidation suggests links between antioxidant consumption and the one-electron oxidation of Trp residues with UVB irradiation.

Given the pivotal role played by one-electron oxidation of Trp in the propagation of photooxidative damage to multiple sites of apoA-I, ApoA-II, and apoB100, it is essential to establish relationships between the initial Trp photoionization step and reactions which repair UVB-induced damage to apolipoproteins.

The investigation of kinetics in such reactions requires the production of ^•^Trp concentrations much greater (~*μ*M) than those obtained under steady-state irradiation (≪pM) with incident light doses comparable to those used in [13, 15, 25]. In HDL and LDL aqueous solutions, micromolar ^•^Trp concentrations can be produced rather selectively by pulse radiolysis [20], a fast kinetics spectroscopic technique involving radiolysis of water with a high energy electron pulse of a few nanosecond duration. Such electrons produce almost equal yields of ^•^OH and *e*_aq_ as major radical species with H^•^ atoms as a minor component. Subsequently, ^•^Trp radicals are formed within 50 *μ*s by reaction of Trp residues with ^•^Br_2_^−^ radical-anions. These radical-anions are selective, mild oxidants formed by scavenging the ^•^OH radicals with Br^−^ anions. Most importantly, ^•^Br_2_^−^ is the sole radical formed in N_2_O-saturated solutions, while both ^•^Br_2_^−^ and ^•^O_2_^−^ radical-anions are simultaneously produced at almost equal yields in air- or O_2_-saturated solutions.

**Figure 5:**
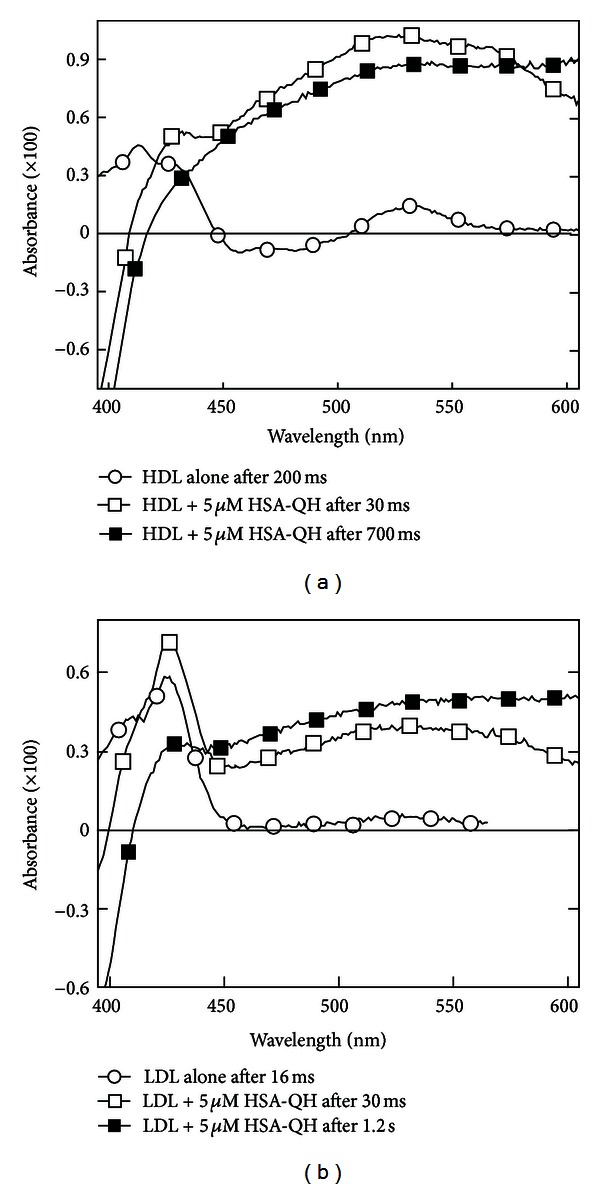
(a) Absorbance of apolipoprotein and quercetin radicals in HDL_3_. (*⚪*) Transient absorbance spectra of 12.5 *μ*M HDL_3_ in N_2_O saturated 10 mM pH 7 phosphate buffer containing 0.1 M KBr recorded 200 ms after oxidation with 3.2 *μ*M of ^•^Br_2_^−^ radical-anions. (□, ■) The same but solutions contained 18.75 *μ*M HDL_3_, 5 *μ*M HSA, and 5 *μ*M QH. Spectra were recorded at 30 ms (□) and 700 ms (■) after oxidation with 2.9 *μ*M of ^•^Br_2_^−^ radical-anions. (b) Absorbance of apoB100 and quercetin radicals in LDL. (*⚪*) Transient absorbance spectra of 1.6 *μ*M LDL in N_2_O saturated 10 mM, pH 7, recorded 16 ms after oxidation with 4.0 *μ*M of ^•^Br_2_^−^ radical-anions. (□, ■) The same but the solutions contained 2.4 *μ*M LDL, 5 *μ*M HSA, and 5 *μ*M QH. Spectra were recorded at 30 ms (□) and 1.2 s (■) after oxidation with 3.2 *μ*M of ^•^Br_2_^−^ radical-anions. Redrawn from data in [32].

Using the pulse radiolysis technique, the transient absorbance parameters, absorbance maximum (*λ*_max⁡⁡_) as well as molar extinction coefficient (*ε*_max⁡⁡_) at *λ*_max⁡⁡_, of the ^•^Trp, TyrO^•^, and *α*-tocopheroxyl (*α*TocO^•^) radicals have been measured in the UV-visible spectral regions. With these parameters kinetics of their formation and/or disappearance have been determined in lipoprotein aqueous solutions on time scales extending from microseconds to seconds. The *λ*_max⁡⁡_ characteristics for ^•^Trp, TyrO^•^, and *α*TocO^•^ radicals are 520 nm, 410 nm, and 430 nm, respectively, while the corresponding *ε*_max⁡⁡_ are 1750 M^−1^ cm^−1^, 2700 M^−1^ cm^−1^, and 7100 M^−1^ cm^−1^, respectively. The *α*TocO^•^ radical absorbance also presents a shoulder at 410 nm with *ε*_max⁡⁡_ = 4500 M^−1^ cm^−1^ [30]. This powerful tool allows one to elucidate and quantify unknown repair reaction pathways as well as to characterize the formation and decay of the various radical species in different lipoprotein microenvironments [20, 31]. Transient absorbance spectra presented in Figures 5(a) and 5(b) allow one to clearly identify the different steps of the repair reactions taking place in N_2_O-saturated LDL and HDL solutions following oxidation with ^•^Br_2_^−^ radical-anions. While the repair of ^•^Trp by Tyr residues with formation of TyrO^•^ radicals takes place on a time scale of a few hundreds of *μ*s [20], TyrO^•^ radicals are repaired by *α*TocOH on longer time scales with formation of the *α*TocO^•^. An unrepaired population of ^•^Trp species remains stable for more than 2.5 s. For the repairable damage, the repair reaction can be schematized as

(1) αTocOH+(apoA-I•,apoA-II• or apoB100•) →αTocO•+(apoA-I,apoA-II or apoB100)

Due to the extensive structural differences between their two apolipoproteins, there are notable disparities concerning the rates of repair in the two lipoprotein particles by *α*TocOH. In oxidized LDL, *α*TocO^•^ radicals formation is already completed after 2.5 ms with no indication of remaining TyrO^•^ transient absorbance (see Figure 5(b)). On the other hand, the rate of repair by *α*TocOH is much slower in oxidized HDL (Figure 5(a)) where absorbance of TyrO^•^ radicals at their 410 nm maximum and that of the *α*TocO^•^ radicals at 430 nm are still visible 2.5 s after the radiolytic pulse [31].

An important structural feature should be noted. Each LDL particle contains several *α*TocOH molecules, whereas on average there is only one *α*TocOH molecule in each 3 to 5 HDL particles. This major discrepancy in the average number of *α*TocOH molecules in LDL and in HDL has two implications. First, there is a much greater intrinsic probability of TyrO^•^ radical repair in the LDL particles containing *α*TocOH, leading to a much faster reaction rate (1) as evidenced by the rapid disappearance of the TyrO^•^ radical absorbance over ~300 *μ*s (Figure 5(b)). Secondly, the observation of TyrO^•^ transient absorbance at least 2.5 s after HDL oxidation by ^•^Br_2_^−^ results from the absence of *α*TocOH in ~60% to 80% of the HDL particles, with no repair being possible in those particles devoid of *α*TocOH (Figure 5(a)). By analogy, it is reasonable to assume that the limited repair of the TyrO^•^ and ^•^Trp radicals in the UVB-induced oxidation of HDL and LDL leads to the permanent damage evidenced by the immunoblots of Figures 2 and 3 and the oxidation of Trp residues (Figure 1). Furthermore, the time scale of the various repair reactions by *α*TocOH and Car described here after one-electron oxidation of Trp residues by ^•^Br_2_^−^ is consistent with the kinetics of bleaching observed with UVB photooxidation.

**Figure 6:**
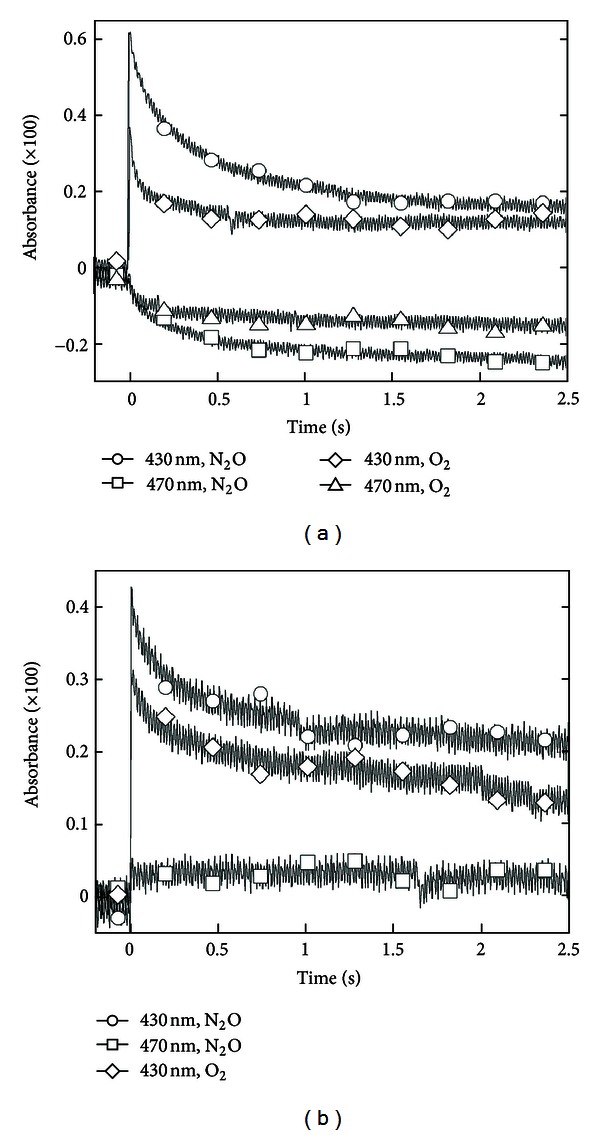
(a) Decay of transient absorbance of *α*TocO^•^ radicals at 430 nm (*⚪*, *⋄*) and bleaching of the carotenoid absorbance at 470 nm (□,  ∆) after oxidation of 20 *μ*M HDL by ^•^Br_2_^−^ radical-anions in 10 mM pH 7 phosphate buffer. Solutions were saturated with N_2_O (□,  *⚪*) and O_2_ (*⋄*,  ∆). (b) Transient absorbance changes measured at 430 nm and 470 nm for solutions containing 1.6 *μ*M LDL. In (a) and (b), [^•^Br_2_^−^] = 3.0 *μ*M for N_2_O-saturated solutions and [^•^Br_2_^−^] = 5.0 *μ*M for O_2_-saturated solutions (see [31] for full experimental details).

Another interesting feature is revealed in Figure 5(b) by comparing transient spectra obtained 30 ms and 2.5 sec after oxidation by ^•^Br_2_^−^ radical-anions. A bleaching is observed in the 440–510 nm region corresponding to Car absorption. Analyses of the kinetics of formation and decay of the *α*TocO^•^ radicals in HDL at 430 nm and of the Car consumption over a 2.5 s time scale (Figure 6) demonstrate a limited (2%) repair of *α*TocOH by Car according to the following reaction:

(2) αTocO•+Car→Car+•+αTocOH

Interestingly, this partial “sparing effect” is only observed in HDL even though LDL contains 10 times more Car than HDL [31]. Comparative studies of LDL and HDL_3_ by surface pressure measurements on monolayers [34] and by EPR with spin labeled fatty acids [35] have demonstrated that the smaller HDL particles (e.g., ~9 nm diameter versus 20 nm for LDL) with lower nonesterified cholesterol and less saturated phospholipid composition have a more fluid structure. As a result of increased fluidity and of the interpenetration of apoA-I within the particle, the full sequence of oxidation and repair reactions can occur.

## 6. Sensitivity of ***α***TocO^•^ Radical Decay and Car Bleaching to the Presence of O_**2**_

The effects of oxygen on radical reactions involved in the formation and repair of oxidative damage in apoA-I and apoA-II or apoB100 have been considered because of the obvious relevance to processes in the *in vivo* environment. The ^•^Trp, TyrO^•^, and *α*TocO^•^ radicals are rather unreactive with oxygen itself. On the other hand, *α*TocO^•^and^•^Trp, in the free form or in proteins, readily react with the ^•^O_2_^−^ radical-anion. Additionally, *α*TocO^•^ can directly oxidize Car but not Trp or *α*TocOH itself (see [31] for key references). However, the complex lipoprotein structure and associated microenvironments modulate this reactivity. Surprisingly, ^•^Trp radicals do not react with ^•^O_2_^−^ in LDL or in HDL suggesting reduced accessibility of the pool of remaining long-lived ^•^Trp radicals to ^•^O_2_^−^ in both lipoproteins.

Figures 6(a) and 6(b) show that the *α*TocO^•^ radical yields resulting from repair of oxidative damage to apoA-I and apoA-II as well as to apoB100 are approximately half those measured under N_2_O saturation; this is consistent with the expected yields of TyrO^•^ and ^•^Trp radicals under O_2_ saturation. They also show that at short times after the radiolytic pulse, a portion of *α*TocO^•^ radical in HDL disappears at an increased rate while the remainder—represented by at least 50% of the absorbance—is hardly affected by the presence of ^•^O_2_^−^ radicals. Thus, from three categories of *α*TocO^•^ species identified in these lipoproteins, only two react with ^•^O_2_^−^ presumably by the following repair reaction:

(3) αTocO•+O2•−+H+→αTocOH+O2

which leads to partial *α*TocOH restoration. Carotenoid bleaching, accounting for partial *α*TocOH repair, is still observed with HDL (Figure 6(a)) but not with LDL in which the lack of Car bleaching rules out direct Car oxidation by ^•^O_2_^−^ and, thus, penetration of ^•^O_2_^−^ into the LDL lipid core.

## 7. Polyphenols as Effective Antioxidants in Repair of Oxidative Damage to Apolipoproteins: Restoration of ***α***TocOH by Albumin-Bound Quercetin

Given the particular role attributed to flavonoid-type antioxidants in the control of atherogenesis [36], it is of apparent interest to determine whether quercetin (QH)—when bound to its physiological carrier, HSA [37]—may supplement the incomplete repair of apolipoprotein damage in HDL_3_ and LDL by endogenous *α*TocOH and may thus ameliorate the subsequent biological consequences of skin irradiation.

The transient absorbance spectra observed thirty milliseconds after the radiolytic pulse in N_2_O-saturated solutions of HDL_3_ and LDL containing 5 *μ*M QH and 5 *μ*M HSA are shown in Figures 5(a) and 5(b). In these figures, the transient absorbance spectrum of the ^•^Q radical, that is, the semioxidized QH molecule, appears as a very broad spectrum extending from the near UV to the far red. The formation of ^•^Q radicals also accounts for the bleaching of the QH absorption in the near UV region. Hence, kinetic analyses may be most conveniently performed in the red region at a wavelength with no contribution from the absorbance of the TyrO^•^, ^•^Trp, and *α*TocO^•^ radicals or from QH bleaching [38].

In the minor fraction of HDL_3_ containing *α*TocOH, the semioxidized species, TyrO^•^ is repaired by endogenous *α*TocOH, generating *α*TocO^•^ radicals. In addition, two populations representing 80% of *α*TocO^•^ initially formed are repaired over a 3-second time scale by quercetin bound to HSA at physiologically relevant concentration. In contrast to the *intramolecular *repair reaction by the endogenous antioxidants leading to *α*TocO^•^ or ^•^Car^+^ formation [31], the HSA-bound ^•^Q radicals are formed by *intermolecular *reaction implying collision between HSA and the lipoproteins.

In the major fraction of HDL_3_ particles lacking *α*TocOH, both TyrO^•^ and ^•^Trp are repaired by free and HSA-bound quercetin. In LDL particles, all of which contain *α*TocOH, *α*TocO^•^ radicals are formed on the millisecond time scale by the repair of TyrO^•^ radicals produced in apoB100. Subsequently, 75% of initial *α*TocO^•^ are repaired by HSA-bound quercetin over a time interval of seconds. In summary, the following repair reactions have been demonstrated:

(4a) TyrO•+QH→Tyr+Q•

(4b) αTocO•+QH→αTocOH+Q•

(4c) Trp•+QH→Trp+Q•

Once the major reactions have been characterized in de-aerated solutions, the intervention of O_2_ in these reactions can be analyzed. First, it should be noted that the ^•^O_2_^−^ radical-anion can readily oxidize QH with high rate constant [32] according to

(5) QH+O2•−+H+→Q•+H2O2

The transient absorbance spectra observed in O_2_-saturated solutions of HDL_3_ and LDL containing 5 *μ*M QH and HSA are similar to those obtained in the absence of oxygen. In addition to the direct reduction of the ^•^O_2_^−^ radicals (5), the reactions (4a), (4b), and (4c) observed in de-aerated solutions also occur. The fraction of *α*TocO^•^ radicals (more than 50%) not repaired by superoxide radical-anions can be repaired by HSA-bound quercetin with formation of ^•^Q but to a much lesser extent in LDL than in HDL [38].

The extensive repair of oxidative damage to lipoproteins by QH is particularly interesting as QH exists in plasma as conjugated derivatives with antioxidant activities comparable to or exceeding that of unconjugated QH [39].

Several recent studies suggest that newly synthesized flavones such as 3-alkyl-3′,4,5,7-tetrahydroxyflavones [28] or hydroxyl-2,3-diarylxanthones [40] are much more effective antioxidants than QH in the Cu^2+^-induced LDL oxidation model [28] or in restoring *α*TocOH [40]. Because of their obvious relevance to dermatology and/or possible cosmetic applications, such new antioxidants merit further evaluation. Unfortunately, it should be recalled that antioxidants—for example, QH—may be either pro- or antioxidant under some experimental conditions in the Cu^2+^-induced LDL oxidation model [29]. It is known that contrasting behaviours may be exhibited among the flavonoid molecules closely related to QH. For example, flavanol catechin, flavonol QH, and the flavones luteolin and rutin effectively protect human skin fibroblasts against the photooxidative stress induced by UVA alone or in the presence of a photosensitizer. By contrast, the isoflavones genistein can aggravate the photodamage [41]. Thus, the efficacy of antioxidants introduced by natural nutrition or supplementation is still under debate [42].

## 8. Putative Biological Consequences of UVB-Induced Lipoprotein Oxidation

The present review illustrates that photooxidation and autooxidation of serum lipoproteins share common mechanisms although they occur on quite different time scales. These involve the direct oxidation of Trp residues with propagation of damage to other residues such as Tyr and induction of lipid peroxidation leading to formation of multiple TBARS. These, in turn, can react with free amino groups of the apolipoproteins. For example, aldehydic end products of lipid peroxidation react with Lys residues responsible for LDL binding to their cell receptors. Furthermore, both oxidation modes lead to consumption of vitamin E and Car, the natural antioxidants that lipoproteins carry in human serum. A major difference between photo- and autooxidation is that HDL is strongly altered by light, owing to its large excess in the interstitial fluid as compared to LDL. As a result, this oxidized HDL cannot properly fulfil one of its most important functions, the protection of LDL from autooxidation [43]. Furthermore, the radical chain reactions of lipid peroxidation produce prostanoids with vasoconstrictive activity and platelet aggregation potency [44].

Beside systemic effects, lipoprotein oxidation induces or impairs numerous crucial cell physiological responses. For instance, we have shown that oxidation of HDL by various sources of oxidative stress decreases cholesterol efflux from human cultured fibroblasts. The reduced ability of HDL to remove intracellular cholesterol pools and to bind to their receptors has been attributed to apoA-I and apoA-II alterations involving Lys and Trp residues [45].

In the case of oxidized LDL—depending on the degree of alterations of the apoB100—either an imperfect recognition by its receptor or a direct scavenging by macrophages is observed. As a result, more or less oxidized LDL cannot precisely regulate cholesterol uptake and synthesis by cells [7, 22]. Additionally, it must be noted that irradiation of cultured human fibroblasts with UVA decreases the uptake and degradation of native LDL [46]. Most importantly, oxidized LDL (and presumably photooxidized HDL) inhibits cell migration [7]. These oxidized species are cytotoxic and can induce apoptosis of normal or tumour cells, probably by transferring radical damage from the lipoproteins to cell targets [47]. This ability to induce apoptosis is consistent with activation and/or inhibition of signalling pathways such as signalling kinases (PKC, MAPK) by oxidized lipoproteins or with the ability of oxidized lipoproteins to enhance the DNA binding activities of transcription factors such as NF*κ*B, AP, and STAT1/3 (see [33] and references therein).

**Figure 7:**
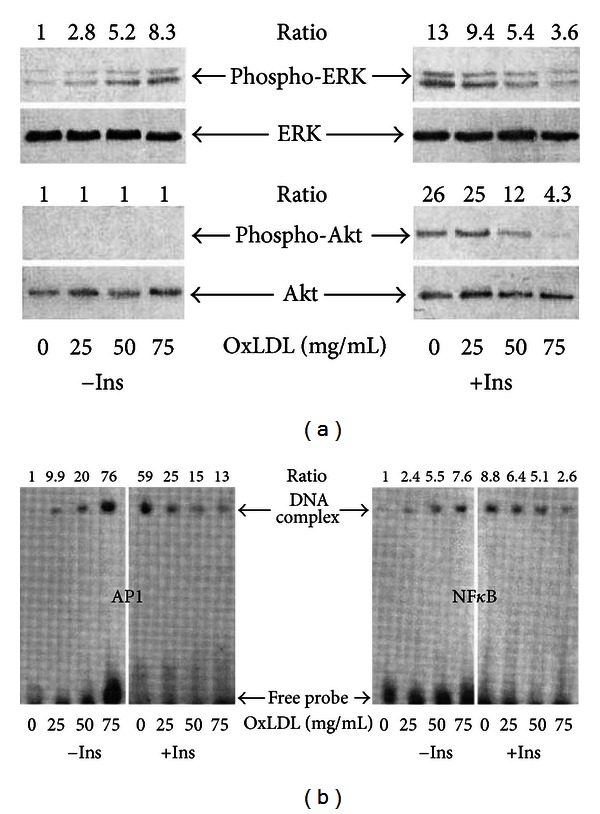
Immunoblots using specific antibodies for ERK, phospho-ERK, Akt, phospho-Akt, and electrophoretic mobility shift assays showing the concentration-dependent effect of oxidized LDL (oxLDL) in presence (Ins) or absence (−Ins) of insulin on signalling kinases ERK and Akt (A) and on transcription factors AP1 and NF*κ*B (see full experimental details in [33]).

The inhibition of insulin (Ins) signalling by oxidized LDL in cultured human fibroblasts is an excellent system to illustrate these properties as these cells have a wide range of biological responses to this hormone [48]. As shown in Figure 7(a), oxidized LDL by itself increases phosphorylation of the signalling kinase ERK but not that of PKB/Akt. In addition, it stimulates the DNA binding activity of AP1 and NF*κ*B transcription factors (Figure 7(b)). Furthermore, oxidized LDL prevents the activation of the Ins-signalling pathway by inhibiting the Ins-induced phosphorylation of ERK and PKB/Akt and the activation of AP1 and NF*κ*B. All these altered signalling events are partially restored by *α*TocOH, demonstrating that the oxidative stress generated by oxidized LDL has a negative effect on the Ins-signalling pathway which is independent of the Ins-induced ROS formation [33].

## 9. Conclusions

The main reasons for the paucity of studies addressing the effects of UVB on the interstitial fluid—as compared to the innumerable ones on skin cells—are probably due to the difficulties in obtaining samples large enough through suction blister fluid collection compared to ready accessibility of skin cells. The analysis of the effects of UVB radiation on major lipoproteins (LDL, HDL) of the interstitial fluid, which bathes the epidermis, demonstrates that to achieve a full understanding of the skin aging process, this medium should not be ignored. Further, the need for such understanding is supported by data which have established the primary processes leading to *α*TocOH and Car consumption after propagation of initial radical damage from Trp residues to other apolipoprotein residues as well as to the lipid core. This review illustrates that, in addition to systemic effects, UVB-induced alterations of proteins of the interstitial fluid may make consequential contributions to inflammation and degenerative processes of skin exposed to UVB attack. Moreover, the local and systemic perturbations reported here concern normal skin. They may also contribute to the mechanism of action and long-term adverse effects observed with the UVB phototherapy of chronic inflammatory skin diseases such as psoriasis and atopic dermatitis [49, 50].
